# Selecting the best optimizers for deep learning–based medical image segmentation

**DOI:** 10.3389/fradi.2023.1175473

**Published:** 2023-09-21

**Authors:** Aliasghar Mortazi, Vedat Cicek, Elif Keles, Ulas Bagci

**Affiliations:** ^^1^^ Department of Computer Vision and Image Analytic, Volastra Therapeutics, New York, NY, United States; ^^2^^ Department of Cardiology, Health Sciences University, Istanbul, Turkey; ^^3^^ Machine & Hybrid Intelligence Lab, Department of Radiology, Northwestern University, Chicago, IL, United States

**Keywords:** deep learning optimization, segmentation, cyclic learning, adaptive optimization, accelerated optimization

## Abstract

**Purpose:**

The goal of this work is to explore the best optimizers for deep learning in the context of medical image segmentation and to provide guidance on how to design segmentation networks with effective optimization strategies.

**Approach:**

Most successful deep learning networks are trained using two types of stochastic gradient descent (SGD) algorithms: adaptive learning and accelerated schemes. Adaptive learning helps with fast convergence by starting with a larger learning rate (LR) and gradually decreasing it. Momentum optimizers are particularly effective at quickly optimizing neural networks within the accelerated schemes category. By revealing the potential interplay between these two types of algorithms [LR and momentum optimizers or momentum rate (MR) in short], in this article, we explore the two variants of SGD algorithms in a single setting. We suggest using cyclic learning as the base optimizer and integrating optimal values of learning rate and momentum rate. The new optimization function proposed in this work is based on the Nesterov accelerated gradient optimizer, which is more efficient computationally and has better generalization capabilities compared to other adaptive optimizers.

**Results:**

We investigated the relationship of LR and MR under an important problem of medical image segmentation of cardiac structures from MRI and CT scans. We conducted experiments using the cardiac imaging dataset from the ACDC challenge of MICCAI 2017, and four different architectures were shown to be successful for cardiac image segmentation problems. Our comprehensive evaluations demonstrated that the proposed optimizer achieved better results (over a 2% improvement in the dice metric) than other optimizers in the deep learning literature with similar or lower computational cost in both single and multi-object segmentation settings.

**Conclusions:**

We hypothesized that the combination of accelerated and adaptive optimization methods can have a drastic effect in medical image segmentation performances. To this end, we proposed a new cyclic optimization method (*Cyclic Learning/Momentum Rate*) to address the efficiency and accuracy problems in deep learning–based medical image segmentation. The proposed strategy yielded better generalization in comparison to adaptive optimizers.

## 1. Introduction

Optimization algorithms are used in the training phase of deep learning, where the model is presented with a batch of data, the gradients are calculated, and the weights and biases are updated using an optimization algorithm. Once the model has been trained, it can then be used for inference on new data.

Stochastic gradient descent (SGD) algorithms are the main optimization techniques used to train deep neural networks. These algorithms can be divided into two categories: adaptive learning rate methods (e.g., Adam and AdaGrad) and accelerated schemes (e.g., Nesterov momentum). Both the learning rate (LR) and momentum rate (MR) are important factors in the optimization process. LR, in particular, is a key adjustable parameter that has been extensively studied and modified over the years. The momentum term was introduced to the optimization equation by Rumelhart et al. in 1986 to allow for larger changes in the network weights without causing oscillation (1).

There have been controversial results in the literature about the characteristics of available optimization methods. Therefore, there is a need for exploring which optimization method should be chosen for particular tasks. Most neural network optimizers have been evaluated and tested on classification tasks, which have much lower output dimensions compared to segmentation tasks, which have much higher output dimensions. Hence, these differences between classification and segmentation problems imply a different investigation and method for optimization. In this paper, we develop a new optimization method by exploring LR and MR optimizers for medical image segmentation problems for the first time in the literature. Our proposed optimizer is simple and promising because it fixes the problems with traditional optimizers and demonstrates how a simple new formulation can solve surprisingly these problems.

### 1.1. Non-adaptive vs. adaptive optimizers

SGD is the dominant optimization algorithm in deep learning, which is simple and performs well across many applications. However, it has the disadvantage of scaling the gradient *uniformly* in all directions (for each parameter of network). Another challenge in SGD is to choose an appropriate value for LR. Since LR is a fixed value in SGD-based approaches, it is critical to set it up appropriately since it can directly affect both the convergence speed and prediction accuracy of neural networks. There have been several studies trying to solve this problem by adaptively changing the LR during training, which are mostly known as “adaptive optimizers.” Based on the history of changes in gradients during network optimization, LR is adapted in each iteration. Examples of such methods consist of *ADAM* (2), ADAGrad (3), and RMSProp (4). In general, adaptive optimizers make training faster, which has led to their wide use in deep learning applications.

The development of momentum in neural network optimizers has followed a similar trajectory as the learning rate. Momentum optimizers (5) were introduced to speed up convergence by considering the changes from last iteration with a multiplier, which is called *momentum*, in updating parameters in current iteration. Selecting an appropriate value for the MR was initially difficult, but this issue was addressed with the introduction of adaptive optimizers like ADAM, which can adaptively adjust both the MR and LR. These adaptive optimizers have become very popular in the field because they quickly converge on training data.

Although they are widely used, adaptive optimizers may converge to different local minima compared to classical SGD approaches, which can lead to worse generalization and out-of-sample performance. This has been demonstrated by a growing number of recent studies (6–8). To improve the generalization ability of neural networks, researchers have returned to using original SGD approaches but with new strategies for improving convergence speed. For example, the *YellowFin* optimizer demonstrated that manually tuning the learning rate and momentum rate can lead to better results than using the *ADAM* optimizer (8). Although it was a proof-of-concept study that provided evidence for the counter-intuitive idea that non-adaptive methods can be effective, however, in practical applications, manually tuning these rates is challenging and time-consuming.

In another attempt, a cyclic learning rate (CLR) was introduced by Smith (7) to change the LR according to a cycle (i.e, triangle or Gaussian), proposing a practical solution to hand-tuning requirements. The CLR’s only disadvantage was that a fixed MR could limit the search states of LR and MR and cause them to fail until finding an optimal solution. Our work will go beyond this constraint.

### 1.2. Summary of our contribution

With motivation from the study by Smith (7), here we introduce an improved version of CLR, called “Cyclic Learning/Momentum Rate” (CLMR). This new optimizer alternates the values of the LR and MR during the training, which has two benefits compared to adaptive optimizers. First, it is computationally more efficient. Second, it has better generalization performance. Furthermore, *CLMR* leads to better results than conventional approaches such as SGD and CLR. Finally, we investigate the effect of changing the frequency of cyclic function in training and generalization and suggest the optimum frequency values. We investigate several optimizers commonly used in medical image segmentation problems and compare their performance as well as generalization ability in single and multi-object segmentation settings by using cardiac MR images (Cine-MRI).

The rest of the paper is organized as follows. In Section 2, we introduce the background information for neural network optimizers, their notations, and their use in medical image segmentation. In Section 3, we give the details of the proposed method and network architectures on which segmentation experiments have been conducted. Experimental results are summarized in Section 4. Section 5 concludes the paper with discussions and future work.

## 2. Background

### 2.1. Segmentation architectures

Over the past few years, there has been a dramatic increase in the use of convolutional neural networks (CNN) in computer vision and medical imaging applications; particular attention is focused on the U-Net style segmentors and, more recently, combined with Transformers (9–13). Here, we briefly review the mostly used segmentation architectures and their characteristics, and choose common baselines for our current study.

In a foundational work detailed by Ciresan et al. (14), the authors embarked on an innovative journey to explore semantic segmentation through the lens of deep neural networks. By employing a convolutional encoder, the authors translated input images into a latent representation, preserving intrinsic characteristics essential for pixel-level understanding. However, their architecture’s decision to leverage fully connected layers manifested a critical limitation. While aiming for fine-grained pixel prediction, these layers inadvertently obfuscated the spatial information integral to the image structure. The consequent degradation in performance was both a challenge and a learning curve, catalyzing further research and refinement in our approach. This key observation not only uncovered essential insights but also paved the way for subsequent advancements in the field.

In a later work, Long et al. (15) introduced the transformative concept of fully convolutional networks (FCNs) as a remedy to the spatial information loss. Ingeniously architected, the FCN employs a sequential cascade of convolutional blocks on the encoder path, each comprising convolution, activation, and pooling layers. This architecture captures the semantic essence of an image, preserving the nuanced details. The elegance of the FCN’s design lies in its decoding path. Employing a series of convolutional layers and up-sampling operations, it reinvigorates the spatial dimensions lost in the encoding phase. This progressive expansion of spatial understanding culminates in fine-grained segmentation results. This innovation by Long et al. heralded a new era in semantic segmentation, deftly balancing complexity with precision and illuminating a path toward a more refined visual comprehension.

The origin of the famous U-Net model comes from the FCNs and the encoder–decoder models. Ronneberger et al. developed the U-Net (16) model for biomedical image segmentation in particular and applied that in a variety of modalities, including CT (17–20), MRI (21–26), US (27, 28), and PET (29, 30). U-Net operates through a dual pathway. This first phase (contracting path) uses convolutional blocks and a downsampling module to encapsulate the essential themes of the image, much like summarizing the chapters of an intricate novel. The second phase (expanding path) magnifies spatial resolutions, often doubling them, to distill the critical elements to a pixel-level classification. One of U-Net’s most notable innovations is the skip connections, a revolutionary design that creates a seamless thread between the contracting and expansive paths. This ensures that vital high-resolution information is not lost in translation but rather reused to fortify the detailed picture with rich context. In essence, U-Net transcends the ordinary, offering a new perspective in medical imagery, where efficiency meets elegance in delivering unparalleled insights.

Many available algorithms mentioned above and recent ones are based on the U-Net kind of architectures or encoder–decoder style approaches. One of the first works in this category was done by Badrinarayanan et al. (31), called *SegNet*, and it was designed for semantic segmentation utilizing an encoder–decoder strategy. Newer U-Net-based algorithms often enhance the backbone architecture designs, bottleneck designs, or architectural engineering to extract richer features. There are a large number of U-Net-based architectures. For example, *ResNet* (32) and *DenseNet* (33) (and their improved versions) have been used extensively as backbones of the U-Net as well as rich feature representation. In the case of *DenseNet*, its unique design of connecting each layer to every other layer in a feed-forward fashion ensures maximum information flow between layers. This structure encourages feature reuse and strengthens gradient flow, which can be particularly useful in the encoder part of the U-Net for capturing intricate patterns. *ResNet*, on the other hand, is known for its residual blocks that allow the network to skip certain layers during training. This skipping mechanism helps in mitigating the vanishing gradient problem, allowing for deeper networks. When integrated into U-Net, *ResNet* can enable the training of deeper models, thus capturing more complex features in the encoder phase.

By leveraging either *DenseNet* or *ResNet* as encoders, the U-Net can benefit from their particular strengths. *DenseNet* can improve the information flow, whereas *ResNet* can facilitate the training of deeper models. Together, these architectures can be flexibly assembled to create U-Nets of varying complexities and capabilities, allowing researchers and practitioners to tailor the network to specific segmentation challenges. For example, by combining the concepts of *ResNet* and *DenseNet*, a new architecture was introduced by Jégou et al. (34) in a U-Net-shaped architecture to do segmentation. There are many more architectures based on the U-Net style with adaptation from the CNN and Transformers literature; a good review paper on this can be found elsewhere (35).

In our current study, we conducted experiments in three different (mostly used) segmentation architectures to demonstrate the effect of the connections, as explained in the following subsections: (1) Encoder–Decoder, (2) U-Net, and (3) Adaptive U-Net (via DenseNet-based architecture in the encoder of U-Net). For (3), we propose to use two versions; hence, we are using four baselines for our experiments in total. Note that we have dropped the use of U-Net architecture from the DenseNet version (Dense-U-Net may be a correct terminology when DenseNet is used as the backbone in U-Net) to simplify the usage. One may increase the number of architectures for more comparisons, but this is outside the scope of our study.

### 2.2. Optimizers with fixed LR/MR

Optimizing a deep neural network, which is a high-dimensional system with millions of parameters, is one of the most challenging aspects of making these systems more practical. Designing and implementing the best optimizer for deep network training has received much attention in recent decades. These studies mainly address two major issues: (1) making the network training as fast as possible (fast convergence) and (2) increasing the generalizability of networks. SGD optimizers have been the most popular optimizer in deep networks due to their low computational cost and fast convergence. There have been major modifications to the original SGD optimizer during the last decade to increase the efficiency for training deep nets. The following are some of the key optimization studies related to our efforts.

SGD and Mini-batch gradient descent were first optimizers used for training neural networks. The updating rule for these optimizers include only the value of last iteration as shown in Equation 1. Choosing appropriate value for an LR is challenging in these optimizers since if LR is very small then convergence is very slow, and if LR is set high, the optimizer will oscillate around global minima instead of converging:

(1) $$\theta_{i}=\theta_{i-1}-\alpha \nabla_{\theta_{i}}J(\theta_{i}),$$

where θ are network parameters, α is LR, and *J* is the cost function to be minimized [function of θ, X(input), and Y(labels)]. Equation 1 can be considered an updating rule for SGD and mini-batch gradient descent by choosing X and Y as whole samples, a single sample, or a batch of samples in a dataset.

The Momentum optimizer was designed to accelerate the optimization process by taking into account the values from previous iterations, weighted by a factor known as “momentum,” as mentioned by Qian (5). The updating rule for this optimizer is defined as

(2) $$\theta_{i}=\theta_{i-1}-\alpha \nabla_{\theta_{i}}J(\theta_{i})-\beta (\theta_{i-1}-\theta_{i-2}),$$

where β denotes the MR. In the *Momentum* optimizer, the past iterations do not play any role in the cost function, and the cost function is calculated only for the current iteration. Also, similar to LR, choosing a proper value for MR is challenging and it has a correlation with LR too.

*Nesterov accelerated gradient* (36) (NAG) was then introduced to address the limitation of momentum optimizers as well as to accelerate the convergence by including information from previous iterations in calculating the gradient of the cost function as shown in the following equation:

(3) $$\theta_{i}=\theta_{i-1}-\alpha \nabla_{\theta_{i}}J(\theta_{i}-\beta (\theta_{i-1}-\theta_{i-2}))-\beta (\theta_{i-1}-\theta_{i-2}).$$

Compared to optimizers with fixed LR/MR, the NAG optimizer generally shows improved performance in both convergence speed and generalizability.

### 2.3. Optimizers with adaptive LR and MR

A significant disadvantage of optimizers with a fixed LR/MR is that they cannot incorporate information from the gradients of past iterations in adjusting the learning and momentum rates. For instance, they cannot increase the learning rate for dimensions with a small slope to improve convergence or reduce the learning rate for dimensions with a steep slope to avoid oscillation around the minimum point. *AdaGrad* (3) is one of fist adaptive LR optimizers used in deep networks adapting the learning rate for each parameter in the network by dividing the gradient of each parameter by its sum of the squares of gradient, as follows:

(4) $$\theta_{i}=\theta_{i-1}-\alpha \frac{1}{\sqrt{G_{i}+\epsilon }}\circ \nabla_{\theta_{i}}J(\theta_{i}),$$

where $G_{i}$ is a diagonal (square) matrix and each diagonal element equal to the sum of the square of gradient of its corresponding parameters:

(5) $$G_{i}=\sum_{i=1}^{I}(\nabla_{\theta_{i}}J(\theta_{i}){)}^{2},$$

where *I* is the current iteration.

One of the drawbacks of *AdaGrad* is gradient vanishing due to accumulation of all past square gradients in the denominator of Equation 6 during the training. This leads the gradients to converge to zero after several epochs in training. However, *AdaDelta*, *RMSProp*, and *ADAM* optimizers solved this problem by considering a sum of the past samples within a pre-defined window. *ADAM* optimizer’s updating rule uses past squared gradient (as scale) and also like momentum, it keeps an exponentially decaying average of past gradients. Hence, these adaptive optimizers have advantages over the *AdaGrad* by adaptively changing both LR and MR:

(6) $$\theta_{i}=\theta_{i-1}-\alpha_{i}\frac{\beta_{1}\nabla_{\theta }J(\theta_{i-2})-(1-\beta_{1})\nabla_{\theta }J(\theta_{i-1})}{\sqrt{\beta_{2}+\epsilon }}\circ \nabla_{\theta_{i}}J(\theta_{i}).$$

Adaptive learning methods are costly because they are required to calculate and keep all the past gradients and their squares to update the next parameters. Also, the adaptive learning optimizer may converge into different minima in comparison with fixed learning rate optimizers (6–8).

Alternatively, CLR was proposed to change the learning rate during training, which needed no additional computational cost. CLR is a method for training neural networks that involves periodically changing the learning rate during training. As mentioned earlier, the learning rate is typically adjusted according to a predetermined schedule, such as increasing the learning rate from a low value to a high value and then decreasing it back to the low value over a set number of training iterations. The learning rate is then reset and the process is repeated. This can help the optimization process by allowing the model to make larger updates at the beginning of training and smaller updates as training progresses, potentially leading to faster convergence and better model performance (7). Later in Figure 3A, we show how we use CLR in our methodology.

### 2.4. Cardiac image segmentation

Cardiovascular diseases (CVDs) are the leading cause of death worldwide according to the World Health Organization (WHO). CVDs lead to millions of deaths annually and are expected to cause over 23.6 million deaths in 2030 (37). Cine-MR imaging can provide valuable information about cardiac diseases due to its excellent soft tissue contrast. For example, ejection fraction (EF), an important metric measuring how much blood the left ventricle pumps out with each contraction, can be measured with Cine-MRI. To this end, radiologists often manually measure the volume of the heart at the end of the systole (ES) and the end of the diastole (ED) to measure EF. This is a time-consuming process with known inter- and intra-observer variations. Due to its significance in functional assessment of heart, there have been numerous machine learning–based automated algorithms developed in the literature for measuring EF. In this study, we dedicate our efforts for this application due to its importance in the clinic.

There is a considerable amount of research dedicated to the problem of cardiac segmentation from MR or CT images. Since Xu et al. found a correlation between motion characteristics and tissue properties, they developed a combined motion feature learning architecture for distinguishing myocardial infarction (38). In our another attempt, *CardiacNet* (39) proposed a multi-view CNN to segment the left atrium and proximal pulmonary veins from MR images following by an adaptive fusion. The shape before information from deep networks was used to guide segmentation network to delineate cardiac substructures from MR images (40, 41). As previously stated, the literature and methodologies for cardiac segmentation are extensive. Readers are invited to consult the studies by Bizopoulos and Koutsouris (42) and Zhuang et al. (43) for more comprehensive information.

## 3. Methods

**Figure 1:**
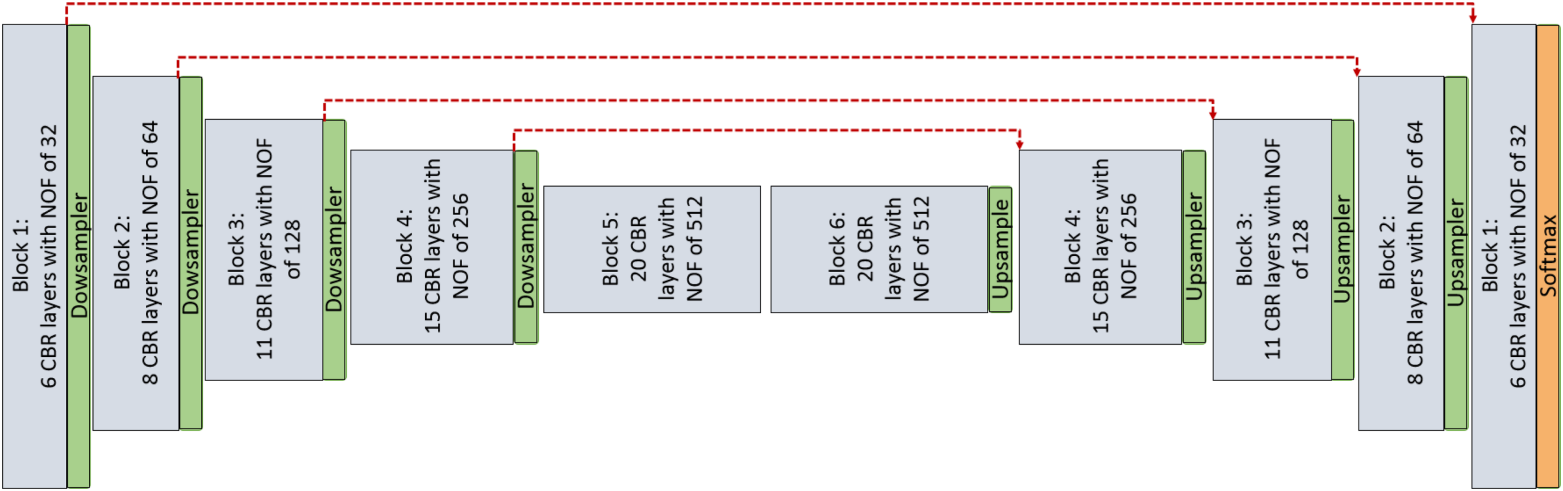
CNN architecture is used for pixel-wise segmentation. The architecture with CNN blocks without red skip connections is the Encoder–Decoder architecture. The architecture with red skip connection (Figure 2A) is called *U-Net*; if connections are with dense block (Figure 2B), it is called Tiramisu (DenseNet for segmentation).

We approach the optimization problem from the perspective of a significant medical image analysis application: *segmentation*. Segmentation is rarely studied from an optimization perspective in comparison to classification. We propose to use four baseline segmentation architectures in our study and assess the optimization procedure in comparison with skip connections, residual connection, or densely connected layers. The medical image segmentation field is so fast growing and there are numerous incremental studies to the following baseline methods, in addition to Transformer-based architectures recently becoming an attractive choice. In our study, our aim is to explore the role and effectiveness of optimization settings; therefore, we are not restricted to any segmentation architectures in our optimization evaluation platform. We hope in this study that we can manage to give the landscape and behavior of the optimization functions, and identify the need for developing efficient ones for computationally heavy segmentation tasks. Baseline CNN architectures used in the experiments are the following:

**1. Encoder–decoder architecture**: This architecture simply consists of the encoder and decoder parts as illustrated in Figure 1, **without** considering red skip connections. The filter size in all the layer are 3×3, and each encoder and decoder part includes five *CNN blocks* and each *CNN block* consists of different number of layers as mentioned in Table 1. Also, the number of filters in each *CNN block* is a fixed number and they are mentioned in Table 1 for each layer. Each layer within the *CNN block* includes *Convolution + Batch normalization + ReLU* as an activation function (*CBR*).

**Figure 2:**
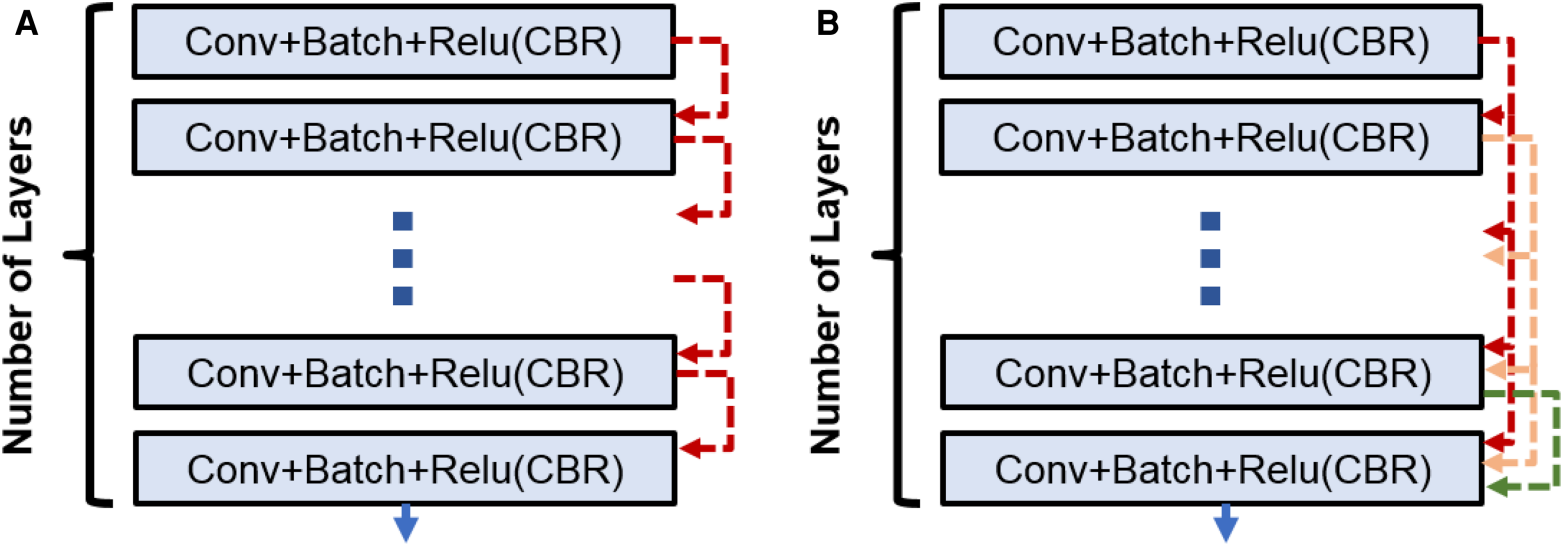
(**A**) CNN block used in Enc–Dec and *U-Net* architectures. (**B**) Dense block used in the Tiramisu architecture.

**Table 1:** Number of layers in each block of different architectures and number of parameters.

	Enc_Dec	U-Net	DenseNet_1 (GR=16)	DenseNet_2 (GR=24)
Block 1	6 layers, #filters=32	6 layers, #filters=32	6 layers	6 layers
Block 2	8 layers, #filters=64	8 layers, #filters=64	8 layers	8 layers
Block 3	11 layers, #filters=128	11 layers, #filters=128	11 layers,	11 layers
Block 4	15 layers, #filters=256	15 layers, #filters=256	15 layers	15 layers
Block 5	20 layers, #filters=512	20 layers, #filters=512	20 layers	20 layers
Block 6	20 layers, #filters=512	20 layers, #filters=512	20 layers	20 layers
Block 7	15 layers, #filters=256	15 layers, #filters=256	15 layers	15 layers
Block 8	11 layers, #filters=128	11 layers, #filters=128	11 layers	11 layers
Block 9	8 layers, #filters=64	8 layers, #filters=64	8 layers	8 layers
Block 10	6 layers, #filters=32	6 layers, #filters=32	6 layers	6 layers
# of params (in million):	77.5	79.1	7.7	8.8

**2. U-Net architecture**: The U-Net model is built entirely from convolutional layers and does not contain any fully connected layers. This makes it well-suited for image segmentation tasks, as it can process input images of any size and output a corresponding segmentation map. The U-Net model is known for its ability to handle small, sparsely annotated training datasets, making it a useful tool for medical image analysis where such datasets are common. This architecture is similar to the Encoder–Decoder architecture as illustrated in Figure 1 **with** red skip connections from the encoder to the decoder. The number of layers and filters for each block are mentioned in Table 1.

**3. DenseNet architecture (adapted into U-Net for segmentation)**: DenseNet is another convolutional neural network architecture that was developed to improve upon the efficiency of training deep networks. The key idea behind DenseNet is to connect all layers in the network directly to every other layer, rather than only connecting each layer to its immediate neighbors as is done in traditional convolutional networks. This allows the network to learn more efficient feature representations and reduces the risk of overfitting. DenseNets have been successful in a number of applications and have achieved state-of-the-art performance on image classification and segmentation tasks. We will use two different *DenseNet* architectures in our segmentation experiments. First, the architecture in Figure 1 with dense blocks (DBs) and skip connections is *DenseNet*_1. Then, in order to use higher growth rate (GR), in *DenseNet*_2, at the end of each block a convolution layer with a kernel size of 1×1 is used to decrease the number of its input filters by **C** rate, which **C** is equal to 2 in this paper. The GR in *DenseNet*_2 increased to 24 (from 16 in *DenseNet*_1) while the number of parameters is decreased (Table 1). The number of CBR layers and also the number of parameters are mentioned in Table 1. Note that we use the terminology of DenseNet here for adapted version of segmentation; original jargon for DenseNet is used only for classification/recognition tasks.

### 3.1. Dense block

Within the DB, a concatenation operation is done for combining the feature maps [through direction (axis) of the channels] for the last three layers. So, if the input to lth layer is $X_{l}$, then the output of lth layer is

(7) $$F(X_{l})=CBR(X_{l}).$$

Since we are doing concatenation before each layer (except the first one), the output of each layer can be calculated only by considering the input and output of first layer as follows:

(8) $$\begin{array}{cc} & F(X_{l})\;=\;F\left(\begin{array}{c} l^{\prime }=l-1\\ ⌢\\ l^{\prime }=0 \end{array}F(X_{l^{\prime }})\right)\;\text{\textbackslash{},for}\;l\geq 1\\ & \;\;\;\text{and}\;l=\{1,2,\ldots ,L,\}, \end{array}$$

where ${}^{⌢}$ is the concatenation operation. In addition, for initialization, $F(X_{-1})$ and $F(X_{0})$ are considered as {} and $X_{1}$, respectively, where {} is an empty set and there are L layers inside the block.

Assuming the number of output features for each layer is $K_{out}$ (channel out) and the number of input features for first layer is $K_{in_{1}}$ (channel in). Then, the feature maps growth (channel out) for second, third, …, and Lth layer are $K_{out}+K_{in_{1}}$, 2$K_{out}+K_{in_{1}}$, …, and $(L-1)K_{out}+K_{in_{1}}$, respectively. The growth rate for the DB is the same as the fourth layer.

### 3.2. Cyclic learning/momentum rate optimizer

**Figure 3:**
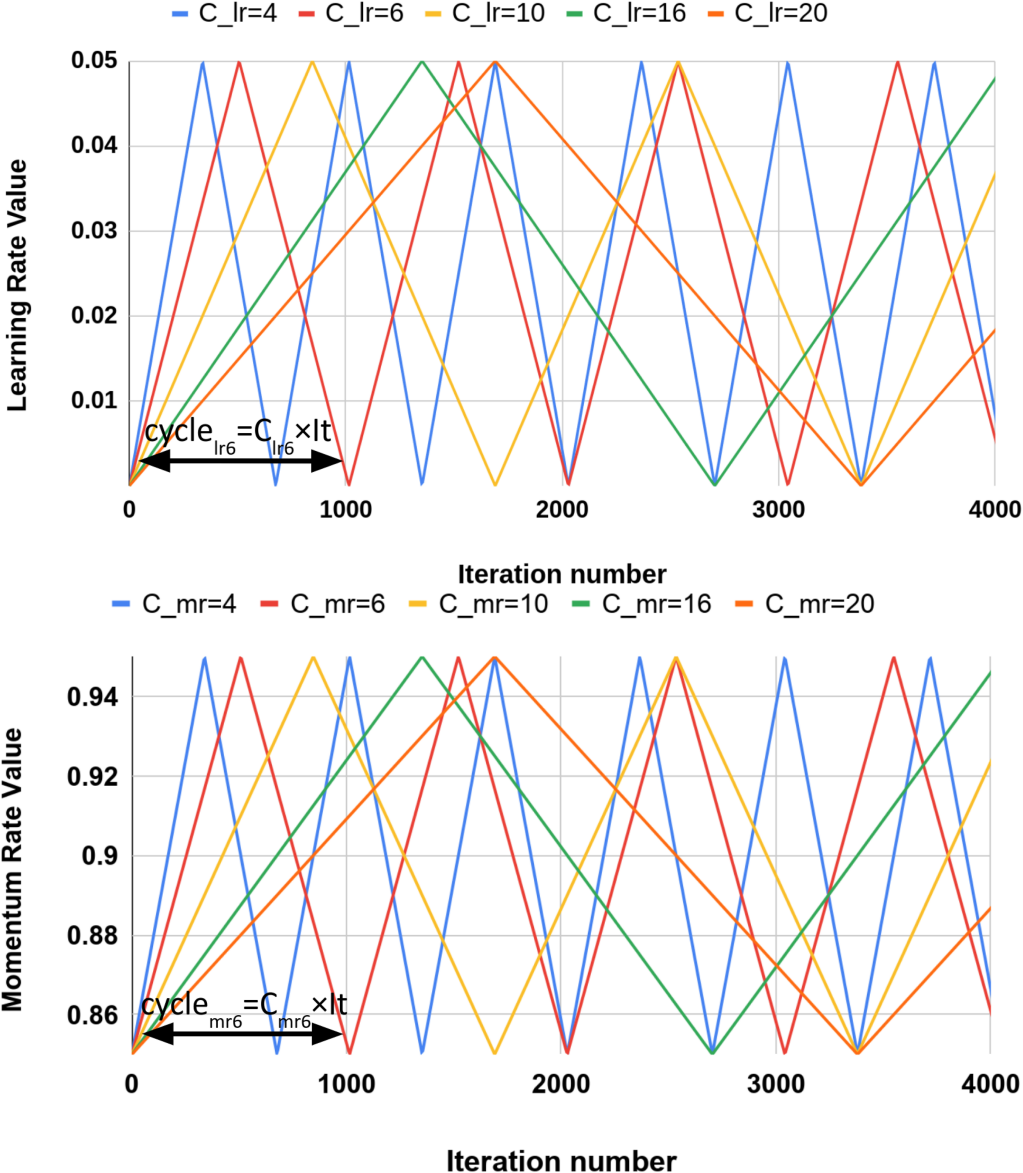
LR and MR functions in cyclic learning setting. (**A**) Learning rate triangle function for different $C_{lr}$ values with $\min_{lr}=0.0005$ and $\max_{lr}=0.05$. (**B**) Momentum rate triangle function for different $C_{mr}$ values with $\min_{mr}=0.85$ and $\max_{mr}=0.95$.

Smith (7) argued that a cyclic learning may be a more effective alternative to adaptive optimizations especially from a generalization perspective. Basically, cyclic learning includes a pre-defined cycle (such as triangle or Gaussian function) in which the learning rate is changing according to that cycle. Here, we hypothesize (and show later in the results section) that having a cyclic momentum in Nesterov optimizer (Equation 2) can lead to a better accuracy in segmentation task in the generalization phase. As a reminder, *momentum* in Equation 2 was used to consider the past iterations by a coefficient called *momentum*. So, choosing the proper value for *momentum* is challenging. To this end, we propose changing the MR in the same way in which we changed the LR, and we considered the cyclic triangle function for both MR and LR as illustrated in Figure 3. $cycle_{lr}$ and $cycle_{mr}$ determine the period of triangle function for LR and MR and are defined as

(9) $$cycle_{lr}=C_{lr}\times It,$$

(10) $$cycle_{mr}=C_{mr}\times It,$$

where $C_{lr}$ and $C_{mr}$ are positive even integer numbers, and *It* is number of iteration per each epoch.

In Figures 3A, B, the cyclic function for different values of $C_{lr}$ and $C_{mr}$ are illustrated. LR during whole training can be determined from Equation 11:

(11) $$LR=\;\left\{ \;\begin{array}{cc} 2\times \frac{\max_{lr}-\min_{lr}}{C_{lr}\times It}\times i+\min_{lr}\;,\; & \;\text{\textbackslash{},for}\;N\times cycle_{lr}\leq i<\frac{2N+1}{2}\times cycle_{lr}\;\\ -2\times \frac{\max_{lr}-\min_{lr}}{C_{lr}\times It}\times i+2\max_{lr}-\min_{lr}\;,\; & \;\text{\textbackslash{},for}\;\frac{2N+1}{2}\times cycle_{lr}\leq i<(N+1)\times cycle_{lr},\; \end{array}\right.$$

where $\max_{lr}$ and $\min_{lr}$ are the maximum and minimum values of LR function, respectively. i is the iteration indicator during whole training process and i∈{1,2,…,It×Ep}, where Ep is the total number of epochs in training and N is a set of natural numbers. MR can also be determined as

(12) $$MR=\;\left\{ \;\begin{array}{cc} 2\times \frac{\max_{mr}-\min_{mr}}{C_{mr}\times It}\times i+\min_{mr}\;,\; & \;\text{\textbackslash{},for}\;N\times cycle_{mr}\leq i<\frac{2N+1}{2}\times cycle_{mr}\;\\ -2\times \frac{\max_{mr}-\min_{mr}}{C_{mr}\times It}\times i+2\max_{mr}-\min_{mr}\;,\; & \;\text{\textbackslash{},for}\;\frac{2N+1}{2}\times cycle_{mr}\leq i<(N+1)\;\times \;cycle_{mr},\; \end{array}\right.$$

where $\max_{mr}$ and $\min_{mr}$ are the maximum and minimum values of the MR function, respectively.

Equations 11 and 12 are used to determine the values of LR and MR in each iteration during training. One of the challenges in using these cyclic LR and MR functions are determining the values of some variables in the equations including $\max_{lr}$, $min_{lr}$, and $C_{lr}$ for LR; and $\max_{mr}$, $\min_{mr}$, and $C_{mr}$ for MR. For finding $\max_{lr}$ and $\min_{lr}$ values, as it suggested by Smith (7), one can run the networks with different LR values for a few epochs and then these values are chosen according to how the network accuracy changes. When both LR and MR change dynamically the one value can affect the other one (considering the optimizer formula), it is more challenging to find the CLMR optimum parameters by the proposed solution. It means we need to train a large number of networks in order to determine the optimum values of $\max_{lr}$, $\min_{lr}$, $\max_{,r}$, and $\min_{mr}$, which is not computationally feasible. Also, a heuristic method was suggested by Smith (7) to find the best value of $C_{lr}$.

In this paper, we propose an alternative way to find best cyclic functions with minimum computational cost. We set fixed values for $\max_{lr}$, $\min_{lr}$, $\max_{,r}$, and $\min_{mr}$ parameters and make sure that the selected values cover a good range for both LR and MR in practice (illustrated in Figure 3). Then, we did a computationally reasonable heuristic search for finding the appropriate amount of $C_{lr}$ and $C_{mr}$ from the values shown in Figure 3. Since changing the values of $C_{lr}$ and $C_{mr}$ leads to change in the values of LR and MR in different iterations, there is no need to find the optimum values for minimum and maximum, and we did search in 2D space of $C_{lr}$ and $C_{mr}$ to find their optimal values.

## 4. Experiments and results

### 4.1. Data

For investigating the performance of proposed method, a dataset from the Automatic Cardiac Diagnosis Challenge (ACDC-MICCAI Workshop 2017) was used (44). This dataset includes 150 cine-MR images: 30 normal cases, 30 patients with myocardial infarction, 30 patients with dilated cardiomyopathy, 30 patients with hypertrophic cardiomyopathy, and the remaining 30 patients with abnormal right ventricle (RV). While 100 cine-MR images were used for training (80) and validation (20), the remaining 50 images were used for testing with online evaluation by the challenge organizers. For a fair validation in training procedures, four subjects from each category were chosen. The binary masks for ground truths of three substructures were provided by the challenge organizers for training and validation while a test set was evaluated online (unseen test set). Three substructures are RV, myocardium of left ventricle(Myo.), and left ventricle (LV) at two time points of ES and ED.

The MRIs were obtained using two MRI scanners of different magnetic strengths (1.5 and 3.0 T). Cine-MR images were acquired with an steady-state free precession (SSFP) sequence in short axis while on breath hold (and gating). In particular, a series of short axis slices cover the LV from the base to the apex, with a thickness of 5 mm (or sometimes 8 mm) and sometimes an inter-slice gap of 5 mm. The spatial resolution goes from 1.37 to 1.68 mm${}^{2}$/pixel and 28–40 volumes cover completely or partially the cardiac cycle.

The use of test, validation, and training data can be summarized as follows. The performance of models during optimization can be monitored using both training and validation datasets. Monitoring the model’s performance on the training data typically allows us to see how well the model is learning from the data it is being trained on. We have used the training data to observe a decreasing loss on the training data until it converges. However, training loss is not a good indicator of the model’s ability to generalize unseen data. Therefore, we used validation data to illustrate our results. By monitoring the model’s performance on a validation dataset, we prevented overfitting too. In our experiments, we made sure that early stopping criteria were used to avoid overfitting, where training loss always decreased to the point where the epochs were stopped. For simplicity, and generalization purposes, we presented the validation loss curves in the experimental results section. Furthermore, we enlisted convergence speed and test error. Accuracy and performance of the results are given on the test data.

### 4.2. Implementation details

The networks were trained for a fixed number of epochs (100) and it was confirmed that they are fully trained; we made sure that all the networks have the same initialization parameters/weights for a fair comparison. All the images were resized to 200×200 in short axis by using B-spline interpolation. Then, as a preprocessing step, we applied anisotropic filtering and histogram matching to the whole dataset. The total number of 2D slices for training was about 1,690 and batch size of 10 were chosen for training. Hence, the number of iteration per epoch is $\frac{1,690}{10}=169$ and we have a total number of iteration 100×169=16,900 in training. The Cross Entropy (CE) loss function was chosen for minimization. All the networks were implemented on Tensorflow with using NVIDIA TitanXP GPUs.

### 4.3. Results

**Figure 4:**
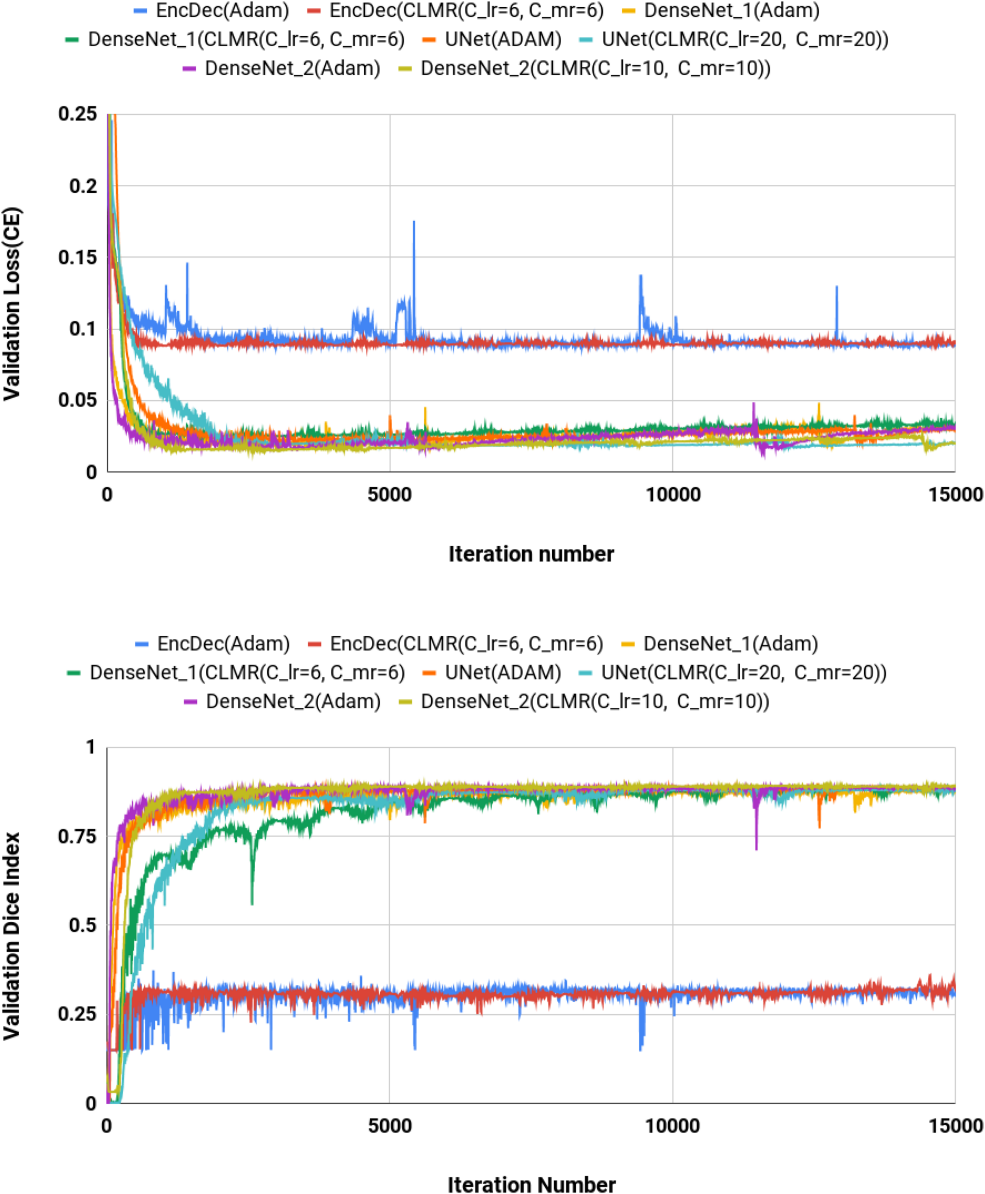
Validation loss and dice index for *DenseNet*_2 architecture with different values of $C_{lr}$ and $C_{mr}$. (Upper) Cross entropy loss in the validation set for *DenseNet*_2 architecture. (Lower) Dice index in the validation set for *U-Net* architecture (zoomed).

**Figure 5:**
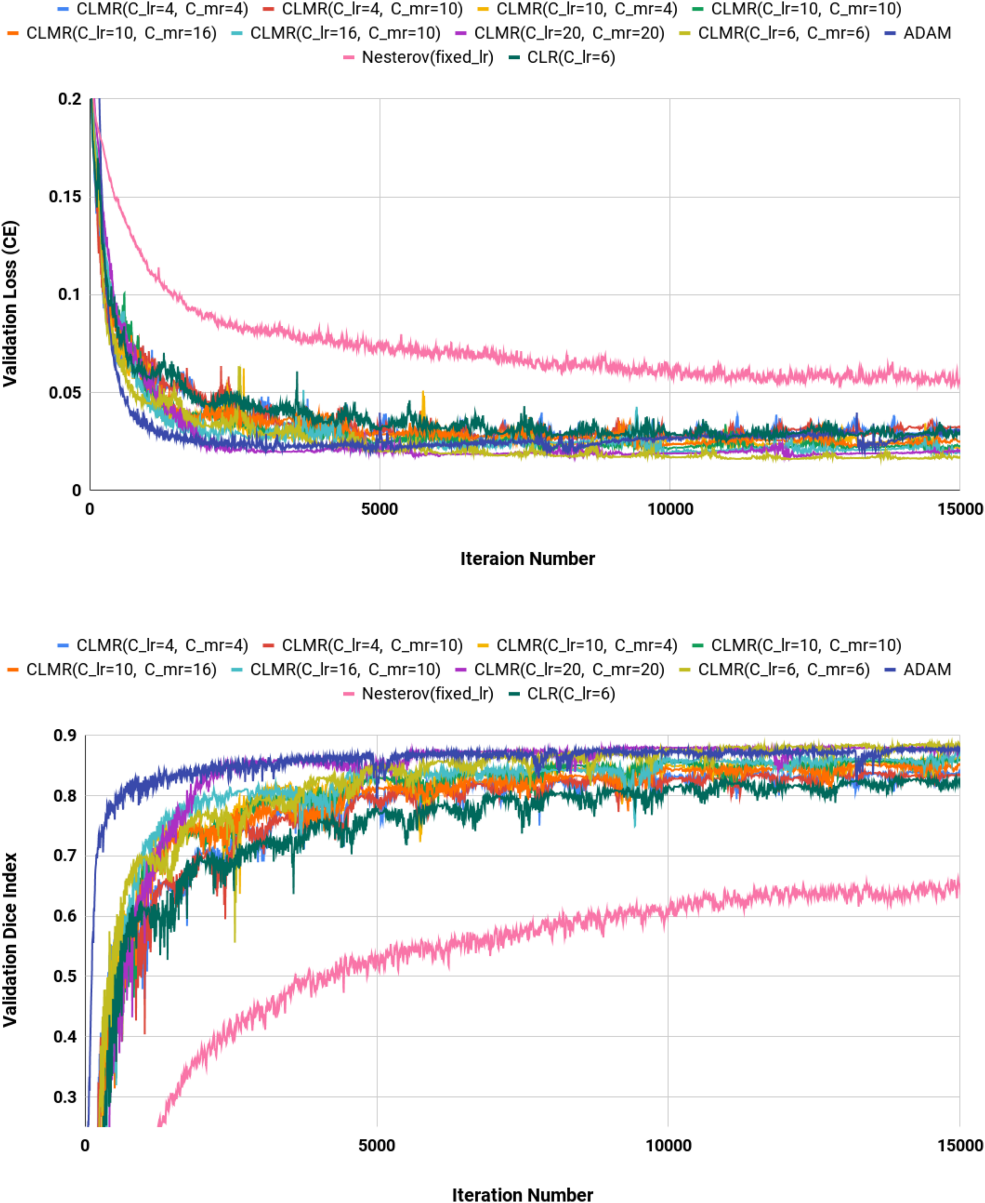
Validation loss and dice index for *U-Net* architecture with different values of $C_{lr}$ and $C_{mr}$. (Upper) Cross entropy loss in the validation set for *DenseNet*_2 architecture. (Lower) Dice index in the validation set for *U-Net* architecture (zoomed).

We calculated dice index (DI) and also CE loss on the validation set for investigating our proposed optimizer along with other optimizers. DI, or dice similarity coefficient (DSC), is a statistical measure used to evaluate the accuracy of the segmentation by comparing the predicted segmentation with the ground truth (the reference standard or manual segmentation). A higher DSC indicates better segmentation accuracy and performance. In Figures 4, the CE and DI curves vs. iterations for *U-Net* architecture for different optimizers are illustrated. As these curves show, the DI in *U-Net* with *ADAM* optimizer is increasing rapidly and sharply at the very beginning and then it is almost fixed afterwards. Although our proposed optimizer [CLMR(C_lr=20, C_mr=20)] is not learning as fast as *ADAM* optimizer at very beginning in *U-Net*, it gets better accuracy than *ADAM* finally. This phenomenon is clearer in CE curves. The quantitative results on the test set in Table 2 support the same observation and conclusion. Furthermore, the same pattern happens for the *DenseNet*_2 architecture in Figure 5. This confirms our hypothesis that adaptive optimizers converge faster but to potentially different local minimas in comparison with classical SGD optimizers.

Figure 4 shows the behavior of the *U-Net* architecture with the *CLMR* optimizer performing 2% increase in dice index (in all three substructures as well as average) than its CRL optimizer counterpart. This proves that having a cyclic momentum rate can yield better efficiency and accuracy than having a simple cyclic learning rate. The results on the test set comparing CLR and *CLMR* optimizers in Table 2 support this conclusion too.

**Table 2:** DI in the test data set with online evaluation.

		Adam	Nesterov	CLR	CLMR
Enc_Dec	*RV*	0.3272	0.1309	0.3833	**0.4336**
U-Net		0.8574	0.5968	0.8618	**0.8820**
DenseNet_1		0.8802	0.6936	**0.8961**	0.8957
DenseNet_2		0.8781	0.7232	0.8910	**0.9049**
Enc_Dec	*Myo*	0.1473	0.1492	**0.1692**	0.1686
U-Net		0.8628	0.6486	0.8588	**0.8631**
DenseNet_1		0.8787	0.7170	0.8834	**0.8960**
DenseNet_2		0.8796	0.7196	0.8904	**0.8999**
Enc_Dec	*LV*	0.4950	0.3260	0.4972	**0.5418**
U-Net		0.9238	0.7670	0.8936	**0.9360**
DenseNet_1		0.9376	0.8465	0.9351	**0.9393**
DenseNet_2		0.9196	0.8449	0.9378	**0.9478**
Enc_Dec	*Ave.*	0.3232	0.1687	0.3499	**0.3814**
U-Net		0.8813	0.6708	0.8714	**0.8937**
DenseNet_1		0.8988	0.7524	0.9049	**0.9103**
DenseNet_2		0.8924	0.7626	0.9064	**0.9176**

Bold values indicate the best performances.

**Figure 6:**
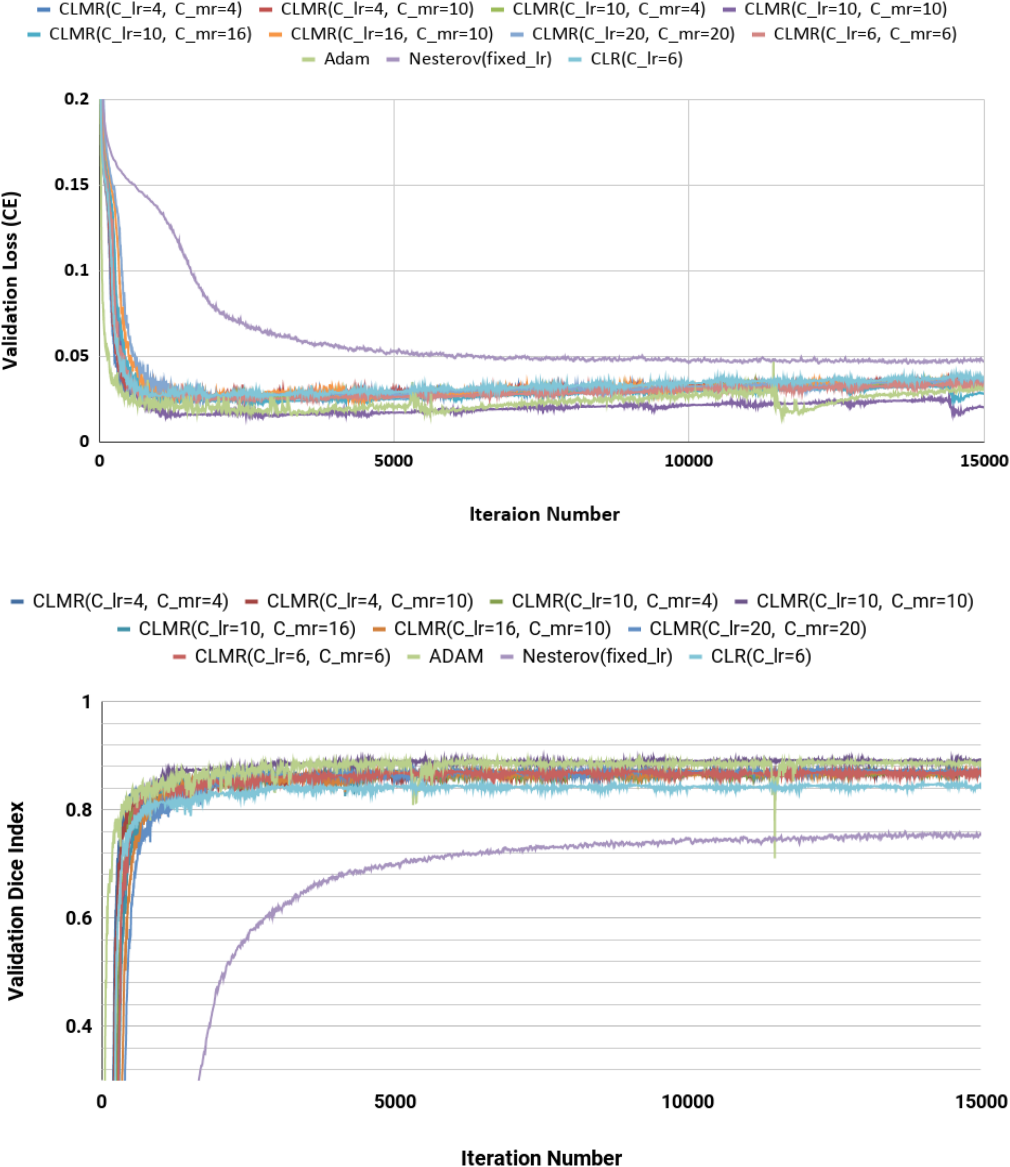
Validation loss and dice index for four different architectures with *ADAM* and *CLMR* optimizers. (Upper) Cross entropy loss in the validation set for four different architectures. (Lower) Dice index in the validation set for four different architectures (zoomed).

Moreover, the curves of DI and CE among different architectures, trained by *ADAM* and *CLMR*, are demonstrated in Figures 6. Although the *DenseNet*_2 has less parameters in comparison with other architectures, it gets better results than the other architectures. These curves reveal some other important points about using different architectures: first, for all different architectures, the proposed *CLMR* optimizer works better than the *ADAM* optimizer, indicating the power of the proposed cyclic optimizer. Second, *DenseNet* architectures are getting better results than *U-Net* and Enc_Dec architectures, which are highly over-parameterized architectures than *DenseNet* and their saturation can be linked to this information too. Third, a comparison between the curves of *DenseNet*_1 and *DenseNet*_2 shows that having a higher GR in dense connections is more important than having a dense block with high number of parameters. *DenseNet*_2, with GR = 24, reached better results in comparison with *DenseNet*_1 with twice of number of parameters in the end of each dense block in comparison to *DenseNet*_2 and GR=16. These results are supported by the dice metric obtained from test data and are mentioned in Table 2.

**Figure 7:**
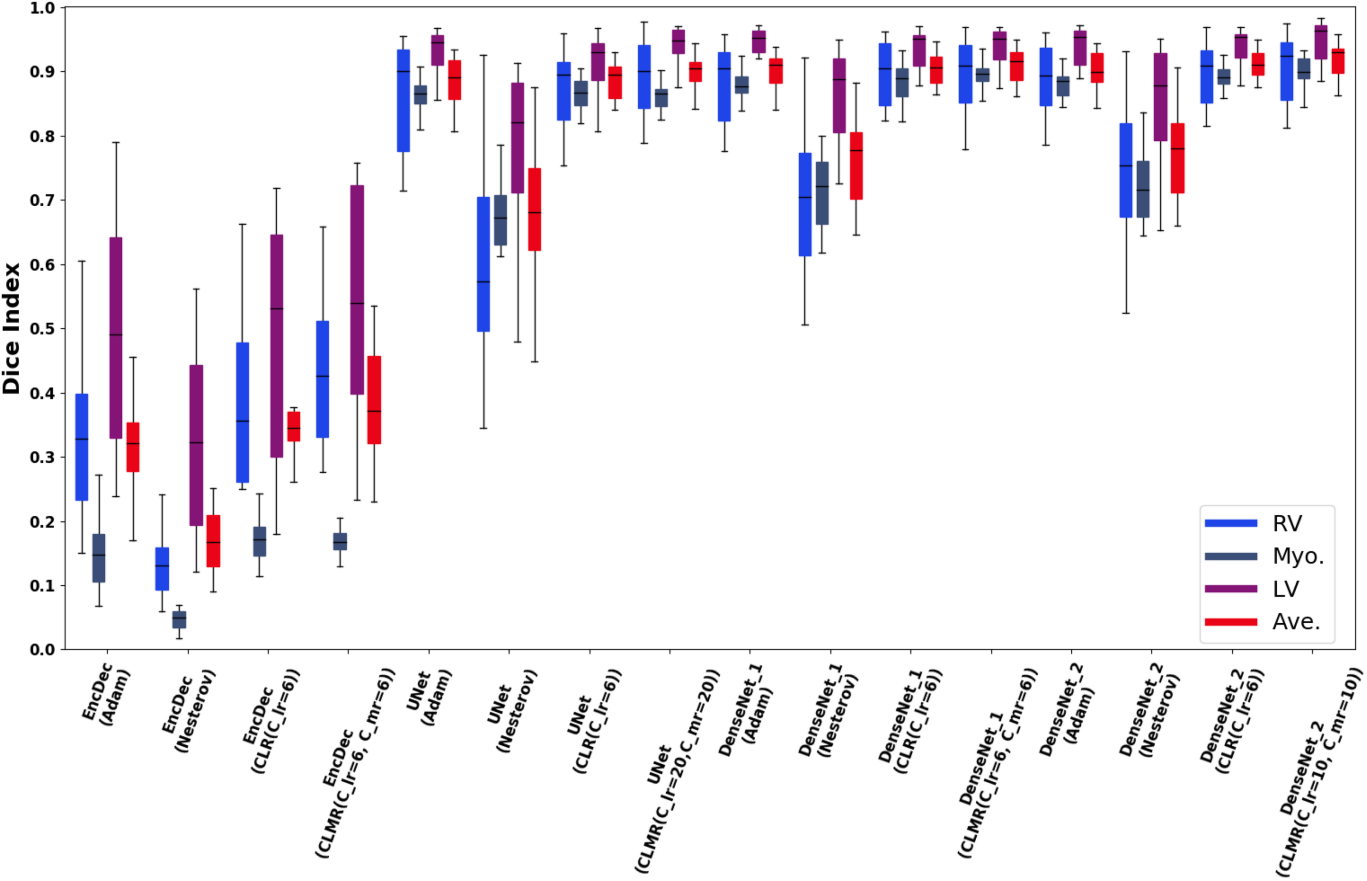
Box plots for DI in test data set for RV, Myo., and LV and their average (Ave).

**Figure 8:**
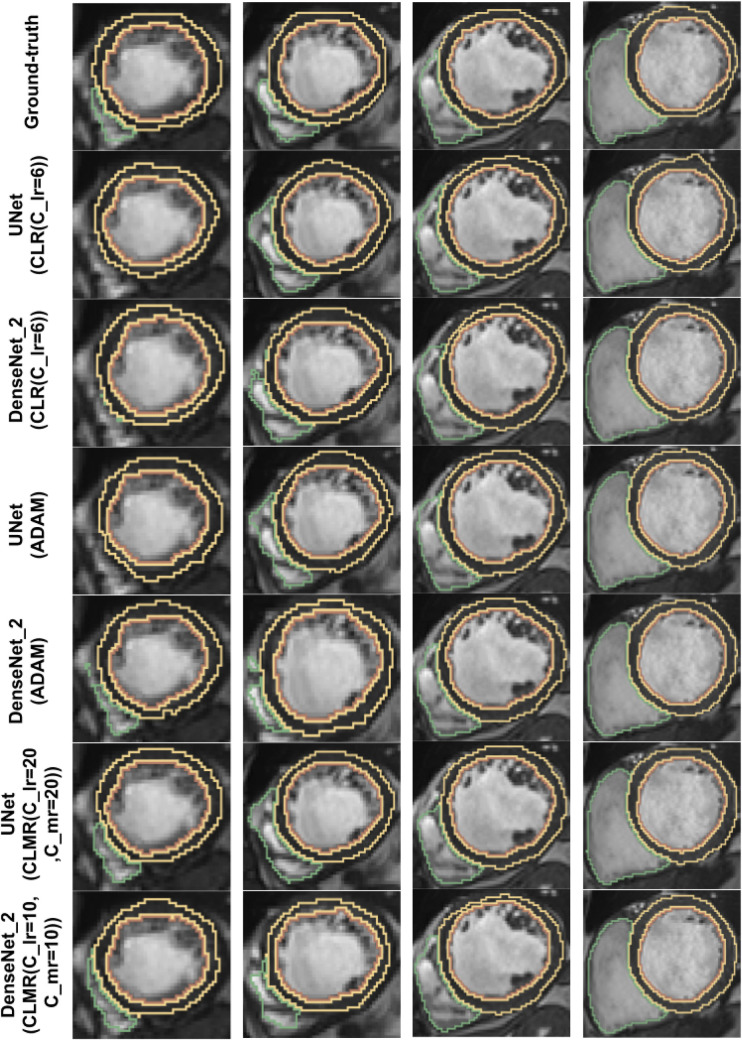
Qualitative results for ground truth and different methods for same subject in end-diastole from Apex to Base for four slices (from right to left). Green, yellow, and brown contours show RV, Myo., and LV, respectively.

**Figure 9:**
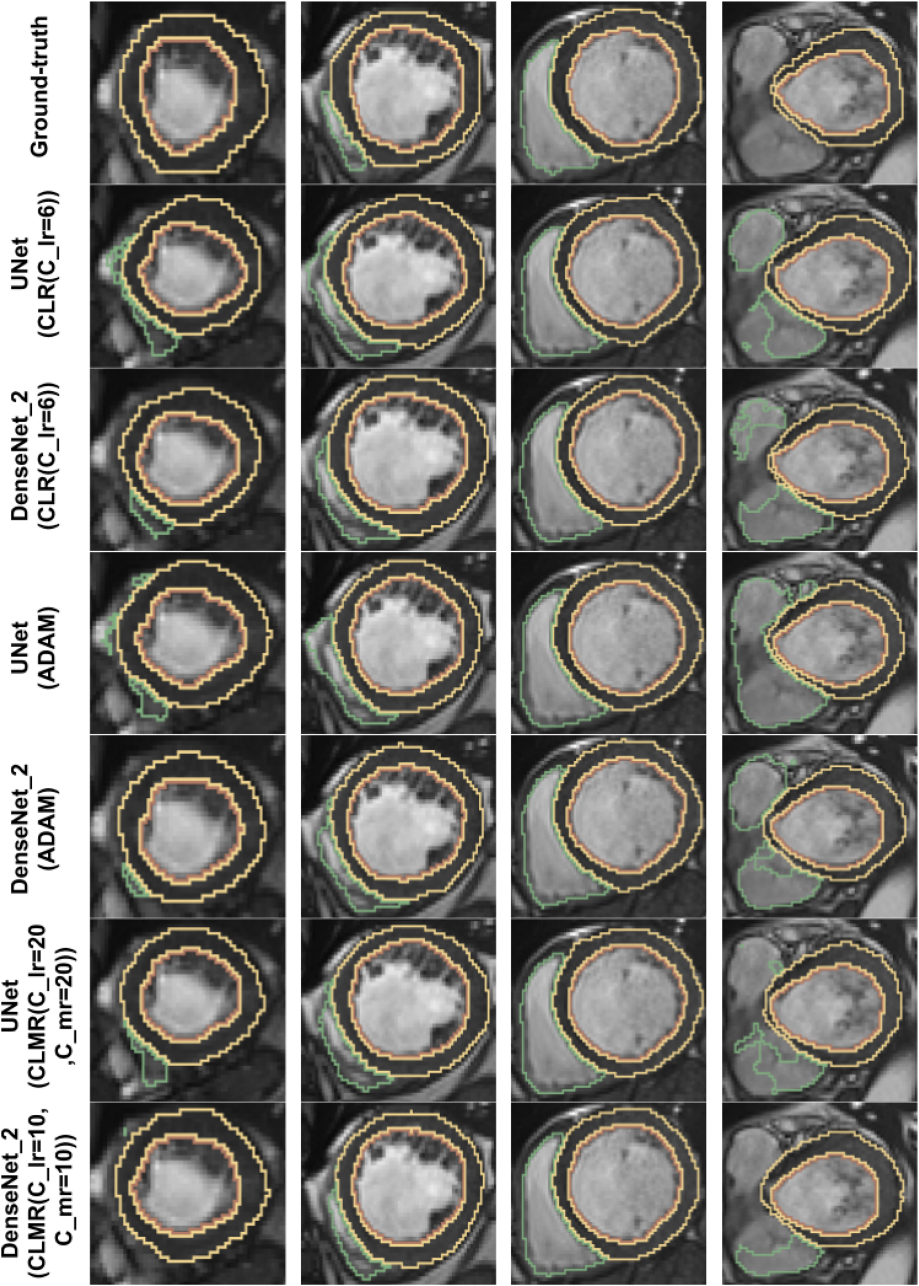
Qualitative results for ground truth and different methods for same subject in end-systole from Apex to Base for four slices (from right to left). Green, yellow, and brown contours show RV, Myo., and LV, respectively.

Finally, the DI on test data with online evaluation for different architectures with different optimizers are summarized in Table 2. In order to have a better comparison, the box plot of all methods are drawn in Figure 7. As the figure shows, the dice statistic obtained from *CLMR* is better than other optimizers most of the time in addition to its superior efficiency. In addition, qualitative results for different methods are shown in Figures 8 and 9: the contours for RV, Myo., and LV in ED for different methods and architectures and also ground truth across four slices from Apex to Base. Usually, segmentation of RV near the Apex is harder than others because RV is almost vanishing at this point. As a result, some methods may not even detect the RV at slices near the Apex. Figure 9 shows the contours for RV, Myo., and LV in ES for different methods and architectures and also ground truth across four slices from Apex to Base. Since heart is at minimum volume at ES, it is more difficult to segment its substructures. The contours generated with the *DenseNet*_2 method is more similar to the ground truth in both ED and ES, which shows the generalizability of the proposed method with an efficient architecture choice.

To ensure reproducibility of the models’ final accuracy in validation and test data, we repeated the experiments for only selected configurations due to the high computational costs associated with rerunning all experiments multiple times. It is important to note that the results obtained from the validation and independent test data sets in the study align with each other. For analysis, we have specifically chosen three best configurations comprising DenseNet-2 with CLMR optimizer, DenseNet-2 with CLR optimizer, and DenseNet-1 with Adam optimizer. These experiments were repeated to compare the average dice index on the validation set and determine if there were any significant differences between them. The resulting P-values for these configurations were 0.805, 0.544, and 0.633, respectively. These P-values indicated that no significant differences were observed between the repeated experiments (P>0.05).

## 5. Discussions and conclusions

We proposed a new cyclic optimization method (*CLMR*) to address the efficiency and accuracy problems in deep learning–based medical image segmentation. We hypothesized that having a cyclic learning/momentum function can yield better generalization in comparison to adaptive optimizers. We showed that CLMR is significantly better than adaptive optimizers by considering momentum changes inside the Nesterov optimizer as a cyclic function. Finding the parameters of these cyclic functions are complicated due to the correlation existing between LR and MR function. Thus, we formulated both LR and MR functions and we suggested a method to find the parameters of these cyclic functions with reasonable computational cost.

Our proposed method is just a beginning of a new generation of optimizers, which can generalize better than adaptive ones. One of the challenges in designing such optimizers is to set up the parameters of cyclic functions that need further investigation in a broad sense. One can learn these parameters with a neural network or reinforcement learning in an efficient manner: i.e., $\max_{lr}$, $\min_{lr}$, $\max_{,r}$, $\min_{mr}$, $C_{lr}$, and $C_{mr}$ can be learned by a policy gradient reinforcement learning approach. In this study, our focus was only on supervised learning methods. However, the proposed method can be generalized to semi-supervised or self-supervised methods as well. This is outside the scope of the current paper and can be thought of as a follow-up to what we proposed here.

In our study, our focus was in a particular clinical imaging problem: segmenting cardiac MRI scans. We assessed the optimization problem with single and multi-object settings. One may consider different imaging modalities and with different, and perhaps with newer, architectures to explore the architecture choices vs. optimization functions. We believe that, based on our comparative studies, the architecture choice can affect the segmentation results such that more complex architectures require optimization algorithms to be selected wisely.

The choice of optimization algorithm can depend on the specific characteristics of the dataset and the model being trained, as well as the computational resources available. Therefore, our results may not be generalizable to every situation in medical image analysis tasks. For instance, if the medical data are noisy or uncertain, it may be more difficult for the model to accurately predict the labels. This can make the optimization process more sensitive to the choice of optimization algorithm and may require the use of regularization techniques to prevent overfitting. For another example, if the dataset is highly imbalanced, with many more examples of one class than the other, it may be more difficult for the model to accurately predict the minority class. This can make the optimization process more challenging and may require the use of techniques such as class weighting or oversampling to improve the performance of the model. Last but not least, if the dataset has a large number of features or the features are highly correlated, it may be more difficult to find a good set of weights and biases that accurately model the data. This can make the optimization process more challenging and may require the use of more advanced optimization algorithms.

Our study has some other limitations too. The use of second-order optimization methods are in high demand recently. However, we did not focus on such methods due to their high computational cost. Second-order optimization methods, which take into account the curvature of the loss function, have shown promising results in a variety of deep learning applications. These methods can be more computationally expensive than first-order methods, which only consider the gradient of the loss function, but may be more effective in certain situations. Furthermore, we focused on the segmentation problem with traditional deep network architectures while reinforcement learning and generative models can require development of new algorithms tailored to specific types of problem.

## Data Availability

Publicly available datasets were analyzed in this study. These data can be found here: MICCAI ACDC 2017 Challenge.
